# Evaluation of China’s Hubei control strategy for COVID-19 epidemic: an observational study

**DOI:** 10.1186/s12879-021-06502-z

**Published:** 2021-08-16

**Authors:** Yu Liu, Fangfang Zheng, Zhicheng Du, Jinghua Li, Jing Gu, Mei Jiang, Daisuke Yoneoka, Stuart Gilmour, Yuantao Hao

**Affiliations:** 1 Department of Medical Statistics and Epidemiology, School of Public Health, Sun Yat-Sen University, Guangzhou, 510080 China; 2 School of Traditional Chinese Medicine Healthcare, Guangdong Food and Drug Vocational College, Guangzhou, 510520 China; 3 National Clinical Research Center for Respiratory Disease, State Key Laboratory of Respiratory Disease, Guangzhou Institute of Respiratory Health, The First Affiliated Hospital of Guangzhou Medical University, Guangzhou, 510120 China; 4 Graduate School of Public Health, St. Luke’s International University, Tokyo, 104-0045 Japan

**Keywords:** COVID-19, Control strategy, Public health interventions, Time-varying effect

## Abstract

**Background:**

To fight against COVID-19, many policymakers are wavering on stricter public health interventions. Examining the different strategies both in and out of China’s Hubei province, which contained the epidemic in late February 2020, could yield valuable guidance for the management of future pandemics. This study assessed the response process and estimated the time-varying effects of the Hubei control strategy. Analysis of these strategies provides insights for the design and implementation of future policy interventions.

**Methods:**

We retrospectively compared the spread and control of COVID-19 between China’s Hubei (excluding Wuhan) and non-Hubei areas using data that includes case reports, human mobility, and public health interventions from 1 January to 29 February 2020. Static and dynamic risk assessment models were developed to statistically investigate the effects of the Hubei control strategy on the virus case growth after adjusting importation risk and policy response timing with the non-Hubei strategy as a control.

**Results:**

The analysis detected much higher but differential importation risk in Hubei. The response timing largely coincided with the importation risk in non-Hubei areas, but Hubei areas showed an opposite pattern. Rather than a specific intervention assessment, a comprehensive comparison showed that the Hubei control strategy implemented severe interventions characterized by unprecedentedly strict and ‘monitored’ self-quarantine at home, while the non-Hubei strategy included physical distancing measures to reduce contact among individuals within or between populations. In contrast with the non-Hubei control strategy, the Hubei strategy showed a much higher, non-linear and gradually diminishing protective effect with at least 3 times fewer cases.

**Conclusions:**

A risk-based control strategy was crucial to the design of an effective response to the COVID-19 outbreak. Our study demonstrates that the stricter Hubei strategy achieves a stronger controlling effect compared to other strategies. These findings highlight the health benefits and policy impacts of precise and differentiated strategies informed by constant monitoring of outbreak risk.

**Supplementary Information:**

The online version contains supplementary material available at 10.1186/s12879-021-06502-z.

## Background

The world is combating the ongoing COVID-19 pandemic, for which potential therapeutics and vaccines are still being investigated. Accordingly, great expectations are placed on non-pharmaceutical public health interventions to contain the epidemic [1, 2]. This has fueled interest in exploring their effectiveness in epidemic control. So far, the potential effects of anti-contagion policies were usually estimated and reported through process-based epidemiological simulations [3–8]. A limited number of control studies based on current and past epidemics focused on the static evaluation of individual or partial interventions [2, 9]. These approaches suffer from a few limitations. First, the information about how long policies should be maintained is obscure or unavailable. Second, the transmission risk in policy scenarios and the interactions between interventions in policy packages are neglected. Therefore, current studies cannot generate an efficient assessment of the time-varying effects of the interventions.

In response to the outbreak of COVID-19 in December 2019, a series of public health interventions has been employed by China’s national, provincial, or municipal governments effectively curbing the epidemic by the end of February. These interventions were triggered by launching the public health emergency response [10, 11]. Besides Wuhan, other cities in Hubei province were recognized as key areas of epidemic growth with city lockdown as milestones for COVID-19 control. Different control strategies were implemented inside and outside of Hubei. This concrete difference in policy interventions ultimately led to different levels of effectiveness. A careful and comprehensive comparison of these two control strategies may help to identify whether or when these interventions should be deployed, consolidated, or relaxed. Such guidance would fill an urgent need in the governmental decision-making process, especially as COVID-19 is still running rampant and policymakers in many countries are wavering on stricter interventions.

Population movement from Wuhan during the 2020 Chinese New Year mass migration constituted an importation risk for the travelers’ destinations [12–14]. In this study, we retrospectively compared the spread and control of COVID-19 in and out of Hubei using data on case reports, human mobility, and public health interventions during the period from 1 January to 29 February 2020. We first characterized the association between the importation risk of COVID-19 and the policy-making process in each study prefecture. Then we documented the difference in control strategies and measures in and out of Hubei province across mainland China. Finally, we developed static and dynamic models to quantify the time-varying effects of Hubei and non-Hubei control strategies.

## Methods

### Data collection

To explore the role of importation risk on control strategy decisions across mainland China and ascertain their impacts, we collected a wide array of data. These data sets included virus case data, control measures implementation details, potentially related population and economic data, and travel data from 16 cities in Hubei province (excluding Wuhan) and 30 provinces outside Hubei. They originated from 46 prefectures in total and ranged in time from the beginning of the COVID-19 epidemic to the time of its fundamental containment (1 January to 29 February 2020). The first three types of data were extracted from local official websites (cases, control measures, and economic/population data). Travel data were retrieved through Baidu Qianxi platform, which derives its data from Location-Based Services. Specifically, Daily Baidu Mobility Indexes (dBMIs) of population outflow from Wuhan to each study prefecture were obtained.

We calculated the total aggregate population outflow from Wuhan between 1 to 26 January 2020 to track movements from Wuhan to each study prefecture before its closure on 23 January 2020 and measure their risks of COVID-19 importation. This data was quantified using the sum of dBMI values, ${x}_{1i}={\sum }_{t=1\mathrm{ Jan}}^{26\mathrm{ Jan}}{\mathrm{dBMI}}_{ti}$ for prefecture *i* and day *t*. We collected data to model the response timing in COVID-19 control strategies. Specifically, for non-Hubei provinces we collected the dates of the Level One emergency response policy (Additional file 2: Table S1). For the cities in the Hubei province, we collected the dates on which the cities initiated shutdown procedures (Additional file 3: Table S2). Additional file 1: S1 provides detailed information about data preparation.

### Data analysis

To analyze the effect generated by the control strategy, we first introduced static models and then dynamic models, which were extended from Jia et al. [12]. The static models generate a cross-sectional analysis of the effect of the control strategy on daily infections and the dynamic models investigate the time-varying effect. Only data after implementing COVID-19 control strategies (26 January) were included for statistical modelling. Because our dynamic models were developed based on a sigmoidal growth pattern of cases, case data with abnormal fluctuations on the epidemic curves were first pre-processed. A variety of events in the early phases of the epidemic lead to abnormal spikes in the number of cases. As more information about the virus surfaced, health professionals amended the diagnosis criteria for the disease (especially for cities in Hubei, Additional file 4: Table S3). Additionally, bulk reporting of jail infections (as happened in Shangdong and Zhejiang) caused other spikes. These anomalies were assumed to be infected several days before and should have been diagnosed and reported earlier than their actual report date. A presumed ought-to-be-reported date was generated for each of them through random assignment. Additional file 1: S2.1 provides a mathematical description for this process.

The static models based on the gravity model [15] have been developed to characterize the effect of population outflow from Wuhan on infections in other prefectures [12]. We extended it to statistically and cross-sectionally investigate the role of the outbreak control strategies. The saturated static model has the following multiplicative exponential form:

$${y}_{i}=c{\cdot e}^{{\sum }_{k}^{3}{\beta }_{k}{x}_{ki}}{e}^{{\lambda }_{1}\cdot {\mathrm{I}}_{Hubei}+{\lambda }_{2}\cdot {D}_{response}+{{\lambda }_{3}\cdot \mathrm{I}}_{Hubei}*{D}_{response}}$$

where ${y}_{i}$ is the cumulative number of confirmed cases in prefecture *i*; ${x}_{1i}$ is the aggregate outflow from Wuhan between 1 to 26 January to prefecture *i*, as described above; ${x}_{2i}$ is per capita GDP; ${x}_{3i}$ is the population density; *c* and ${\beta }_{k}$ are parameters to estimate. ${\mathrm{I}}_{Hubei}$ is an indicator function with ${\mathrm{I}}_{Hubei}=1$ for implementing Hubei strategy, otherwise ${\mathrm{I}}_{Hubei}=0$; ${D}_{response}$ is the response timing (23, 24, or 25 January); ${\mathrm{I}}_{Hubei}*{D}_{response}$ denotes their interaction; ${\lambda }_{j}$ s are the parameters.

A dynamic model under the Cox proportional hazards framework replaces the constant parameters in the static model with a time-varying hazard function ${h}_{0}\left(t\right)$ to model a sigmoidal growth of COVID-19 cases:

$$h\left(t|{x}_{i}\right)={{h}_{0}\left(t\right)e}^{{\sum }_{k}^{3}{\beta }_{k}{x}_{ki}} {e}^{{\lambda }_{1}\cdot {\mathrm{I}}_{Hubei}+{\lambda }_{2}\cdot {D}_{response}+{\lambda }_{3}\cdot {\mathrm{I}}_{Hubei}*{D}_{response}}$$

where $h\left(t|{x}_{i}\right)$ is the hazard function describing cumulative number of confirmed cases at time *t* given other variables ${x}_{i}=\{{x}_{1i},{x}_{2i},{x}_{3i}\}$. Here, the logistic function with parameters $\alpha$, $\gamma$ and $\omega$ was utilized as the sigmoidal growth approximation [16, 17]:

$${h}_{0}\left(t\right)=\frac{\alpha }{1+{e}^{-\gamma t+\omega }}$$

To further improve the model fitting, ${\lambda }_{j}$s are allowed to be time dependent, that is, ${\lambda }_{j}={\lambda }_{j}(t)$. They are empirically determined by the results from the static models.

The R package minpack.lm [18] with a nonlinear least-squares Levenberg–Marquardt (LM) algorithm was used for model fitting and parameter estimation. Models were evaluated using the Bayesian Information Criterion (BIC) and *R*^2^. Additional file 1: S2.2 and S2.3 provide detailed methodologies for this section.

After statistical modelling, the indices of control effectiveness were calculated by exploiting the integral of the differences between predicted and actual case number:

$$IC{E_i} = \sum\limits_{t = 26 {\rm{Jan}}}^{29 {\rm{Feb}}} {\left[ {h\left( {t|{x_i}} \right) - \hat h\left( {t|{x_i}} \right)} \right].}$$

The normalized ${ICE}_{i}$s were used for final measurements of control effectiveness.

## Results

### Importation risk and response timing in each prefecture

**Fig. 1:**
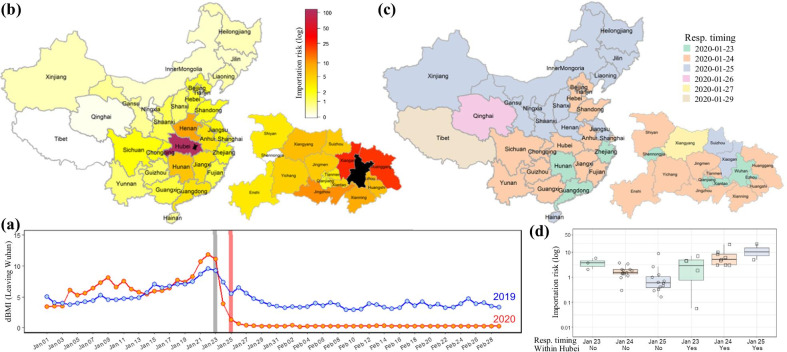
Geographical distribution of the risk of COVID-19 importation to each prefecture and their response timing. **a** Fluxes of population outflow from Wuhan, represented by dBMIs, during period of 1 January to 29 February in 2019 and 2020. The vertical lines in grey and red represent the dates of Wuhan lockdown and Chinese Lunar New Year, respectively. *dBMI* daily Baidu Mobility Index. **b** Spatial distribution of COVID-19 importation risk, measured by their aggregate population inflow from Wuhan until 26 January 2020. Provincial inflow from Wuhan was plotted at the upper left corner and municipal inflow in Hubei plotted at the lower right corner. The black area is Wuhan city. **c** Governments’ response timing in each prefecture. The dates to raise provincial public health response to Level One for COVID-19 control were plotted using different colors (upper left). Similarly, time to execute city shutdown in Hubei was plotted (lower right). **d** The importation risk distribution grouped by governments’ response. Samples with insufficient size at the response timing were excluded, such as Qinghai, Tibet and Xiangyang

In Wuhan, the 2020 Chinese New Year travel rush was interrupted by its lockdown on 23 January and the fluxes sharply declined to almost no movement since 27 January (Fig. 1a). The total aggregate amount of people who entered any study prefecture from Wuhan during this observation period (1–26 January) was used to measure their importation risk (Fig. 1b). More prefectures in Hubei are colored in dark red, indicating high risk of case importation. Comparison using the Wilcoxon Rank Sum test showed that the importation risk to other parts of Hubei is much higher than that to outside provinces with *P* < 0.001 (Additional file 7: Figure S1). Regional differences in risk, both in and out of Hubei, were also observed.

Since 23 January 2020, all the provinces across China successively launched or raised their major public health emergency response to Level One, the highest level (Fig. 1c). Following Wuhan’s outbound traffic closure on 23 January, all other cities in Hubei subsequently announced their shutdown (Fig. 1c). The decline timelines on human mobility had a high consistency with the dates to execute the interventions (Additional file 8: Figure S2). To represent their respective response timing for COVID-19 control, we used the dates of the Level One response launch in non-Hubei provinces and the date of shutdown procedures in Hubei cities (excluding Wuhan). Figure 1d showed that the time to trigger COVID-19 control outside of Hubei was generally consistent with the distribution of the local importation risk with *P* < 0.001 using Jonckheere–Terpstra test. The time to trigger control measures was contrary to the importation risk with *P* = 0.042 for cities in Hubei other than Wuhan.

### Control strategy in Hubei and non-Hubei regions

The major public health emergency response triggered an array of actions by provincial and/or local governments. The main policy instruments deployed included: (1) travel restrictions, (2) case finding and contact tracing, (3) isolation and management of infected individuals and exposed contacts, (4) social distancing, and (5) closed-off community management (Table 1). Despite many similar interventions implemented both in Hubei and non-Hubei areas, important differences remained in control strategies. The control strategy in Hubei required almost all people stay under ‘monitored’ self-quarantine at home. The concrete measures involved unprecedentedly strict closure and traffic restrictions, the ‘monitored’ stay-at-home order, extreme social distancing without any public or business activities, and complete closed-off community management. In contrast, other provinces allowed work resumption from 10 February after application and approval. All resumed enterprises were encouraged to work from home, and businesses’ employees were encouraged to telecommute. The limited enterprises approved to work on-site were required to take rigorous measures to prevent gatherings and cross-infection.

**Table 1:** Comparison between Hubei and non-Hubei control strategy

Measures	Non-Hubei	Hubei
Travel restrictions		
Departure channels from the prefecture through water, land (i.e. road or train) and air transportation	**No**	**Closed**
Water, land or air passenger transport service within prefecture	**No**	**Closed**
Intra-prefecture public transport	**Partially suspended**	**Closed**
Strict traffic control within prefecture, including closure of intra-prefecture highway and shipping, physical isolation and roadblock setup	**No**	**Yes**
Case finding and contact tracing		
Community grid-based screening, e.g. screening and surveillance for people with recent Hubei/Wuhan travel history within the last 14 days	Yes	Yes
Daily health registration and report, e.g. the color-coded health scheme	Required	Required
Routine temperature checking at all places	Yes	Yes
Enhancement of monitoring and online reporting at fever clinics	Yes	Yes
Epidemiological investigation and tracing, e.g. contact follow-up and tracing, then medical observation and nucleic acid testing as needed	Yes	Yes
Isolation and management of infected individuals and exposed contacts		
Isolation and treatment for confirmed cases at dedicated hospitals	Yes	Yes
Quarantine and medical observation for suspected cases at dedicated hospitals	Yes	Yes
A 14-day mandatory quarantine at dedicated facilities for individuals who’ve recently had close contact with someone with COVID-19, and who might have been exposed to COVID-19	Yes	Yes
A 14-day monitored self-quarantine at home or dedicated facilities on individuals who have traveled to the epicenter (e.g. Wuhan or other Hubei area)	**Yes**	**No**
Social distancing		
Extended Spring Festival Holiday	**From 24 Jan to 9 Feb**	**From 24 Jan to 8 Mar**
Public gatherings	Canceled or postponed	Canceled or postponed
The spring semester at school	Postponed	Postponed
Tourist spots and entertainment venues	Closed	Closed
Stay-at-home	**Encouraged**	**Order**
Work resumption	**10 Feb after application and approval**	**9 Mar after application and approval**
Work from home	**Encouraged**	**Not applicable**
Remote commerce	**Encouraged**	**Not applicable**
Strict procedures in essential public facilities (e.g. airports) and enclosed transport vehicles (e.g. planes)	**Yes**	**Not applicable**
Strict procedures in resumed enterprises	**Yes**	**Not applicable**
Government services provided online or through prior reservation	**Yes**	**Not applicable**
Strict health and quarantine measures at points of entry and exit	**Yes**	**Not applicable**
Closed-off community management		
Minimize entrance numbers	Yes	Yes
Set up checking points	Yes	Yes
Issue entry permits	Yes	Yes
Ban non-resident entry	Yes	Yes
Supervise face mask wearing	Yes	Yes
Enhance health monitoring	Yes	Yes
Register personnel and vehicles passing through	Yes	Yes
Shut down community shops	**No**	**Yes**
Execute unified distribution of goods (i.e. grocery delivery) by local community health workers	**No**	**Yes**

The differences between Hubei and non-Hubei control strategy are highlighted in bold

### COVID-19 epidemic trend and association with control strategy

**Fig. 2:**
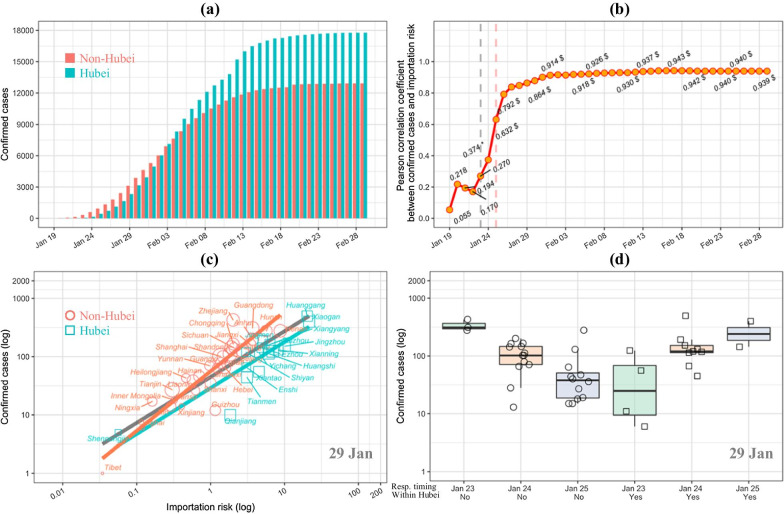
COVID-19 progress and its association with importation risk and control strategy. **a** The epidemic curves of COVID-19 in Hubei and non-Hubei area by 29 February 2020. **b** Relationship over time between the number of confirmed cases (cumulative until 29 February 2020) and total population inflow (up to 26 January 2020) from Wuhan, both on a logarithm scale. **c** The relationship between the log-transformed importation risk (the total population outflow from Wuhan up to 26 January 2020) and the log-transformed number of confirmed cases by prefectures on 29 January 2020. Circles are prefectures in Hubei; rectangles are prefectures outside Hubei; and the point sizes are proportional to the population density of the prefecture. The linear fitting is done for overall (black), Hubei (red) and non-Hubei (cyan) data. **d** The distribution of confirmed cases on 29 January 2020, grouped by governments’ response including response timing and response strategy on a logarithm scale. Samples with insufficient size at the response timing were excluded, such as Qinghai, Tibet and Xiangyang

As of 29 February 2020, a total of 30,702 cases of COVID-19 were confirmed in mainland China excluding Wuhan (Fig. 2a). Among of them, 17,785 (58.9%) cases were reported in Hubei (excluding Wuhan) and 12,917 (42.1%) outside Hubei. These small numbers of cases, relative to tens of millions of people in Hubei or more than 1 billion people outside the province, suggest that the control policy had been working for the epidemic. Specifically, most prefectures experienced a typical sigmoidal growth in cases. This growth was characterized by an accelerated increase in the beginning and a flat period after mid-February (Additional file 9: Figure S3). As expected, Hubei experienced a more serious epidemic characterized by more rapid growth in infections. Abnormal fluctuations were detected in Shandong, Zhejiang, and cities in Hubei, as described in “Methods” section.

Despite the strong association between case progress and importation risk (Fig. 2b), the daily total number of cases in the Hubei area tended to split apart from those in provinces outside Hubei. The overall fit line for all study prefectures highlights this split between the Hubei and non-Hubei areas taken separately (Fig. 2c). Further analysis confirmed this split pattern was maintained over time (Additional file 10: Figure S4a, b). The infectious cases grouped by governments’ responses showed a similar V-shape distribution to their importation risk (Fig. 2d and Additional file 10: Figure S4c, d). These data reveal the potential effects of the intervention control strategies.

### The static and dynamic models for quantifying the effect of control strategy

The preprocessing of case data with abnormally reported dates generated smooth epidemic curves (Additional file 11: Figure S5). In subsequent modelling, the cumulative Wuhan population inflow was always included because of its strong correlation with new daily infections. Each prefecture’s GDP and population density were excluded because statistical tests provided no evidence of correlation (*P* > 0.05).

The static models including aggregate inflow population from Wuhan (before 26 January) and the control strategy as independent variables generated the consistently negative estimates of the coefficients on the Hubei strategy (Additional file 5: Table S4). This finding indicates that the implementation of the Hubei strategy was a continuous protective factor (all *P* < 0.05) in contrast with the non-Hubei strategy. Its protective effects declined over time and remained at a stable level in late February (Additional file 12: Figure S6a). It is noteworthy and significant that the *R*^2^ increased more at the accelerated growth period of COVID-19 cases after introducing the control strategy (Additional file 12: Figure S6b). This observation implies it contributed more during the earlier dates of the epidemic. Subsequently, the response timing was added but no overall effect was found (all *P* > 0.05). When the interaction between the control strategy and response timing was added, the effect of response speed on the epidemic became statistically significant before early February (*P* < 0.05) and all had negative slope estimates (Additional file 5: Table S4). This suggests that the response timing in non-Hubei area may be a ‘risk’ factor during the earlier stage in the implementation of the control strategy. Early implementation of control measures triggers better detection of actual cases, and this should not be considered a ‘risk’ from a policy perspective. This point is further addressed in “Discussion”.

**Fig. 3:**
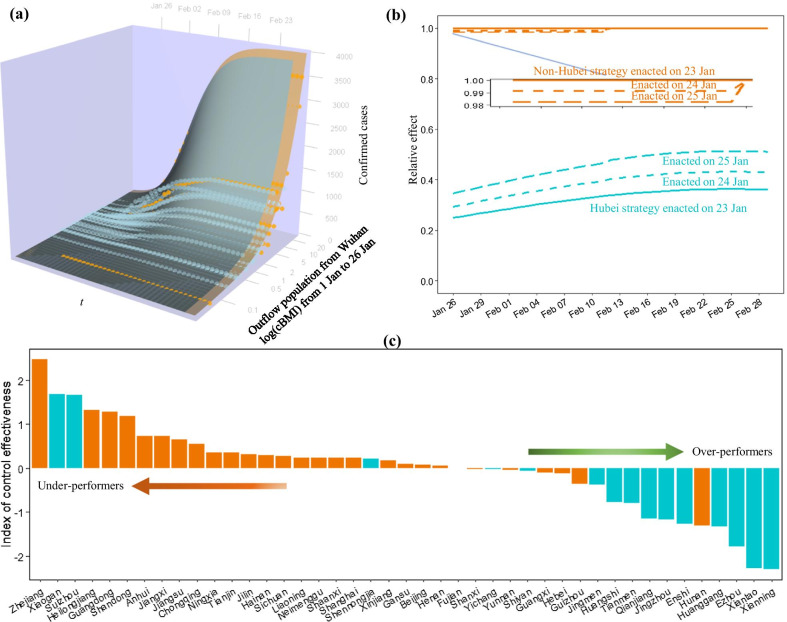
Estimation of time-varying effect of Hubei control strategy. **a** The fitted performance of our dynamic model (see dynamic model I in Additional file 1: Section S2.3). **b** Change of estimated relative risk over time, comparing between Hubei control strategy and non-Hubei control strategy (as reference). **c** Comparison of index of control effectiveness between all study prefectures

The dynamic model using two variables of total population outflow from Wuhan (during 1 to 26 January) to each prefecture and the control strategy showed *R*^2^ = 0.910 (Fig. 3a); and the inclusion of the response timing increased *R*^2^ to 0.922 (Additional file 6: Table S5). According to the features of the coefficients from the static models, we also introduced a quadratic function of time *t* for the variable of the control strategy and a truncated function of time *t* with the cut-off *T* for the response timing. As expected, the BIC improved and *R*^2^ further increased to 0.938 (Additional file 6: Table S5). The truncation date *T* was fixed on 10 February based on the BIC statistics.

Finally, the time-varying effect of the control strategy estimated by the dynamic model with time-dependent effects is shown in Fig. 3b. We used the non-Hubei strategy for COVID-19 control enacted on 23 January as a reference over time. The response timing was unexpectedly shown to be a weak risk factor at about 2 weeks after its implementation. In contrast, the Hubei control strategy showed a very strong protective effect and the protective effect rapidly declined if the implementation was delayed. Precisely, the Hubei control strategy showed a 4 times greater protective effect than the non-Hubei strategy on 26 January if both were taken on 23 January. Despite a narrowing effect, the Hubei area achieved about 3 times fewer cases than non-Hubei areas in late February. The 2-day delay of the Hubei strategy finally narrowed down the effect to 2 times fewer cases. The marginal analysis showed that the Hubei control strategy had the marginal effect curve equal to its effect trajectory enacted on 23 January (Fig. 3b). Overall, the Hubei strategy for COVID-19 control would achieve 3 times fewer cases than the non-Hubei strategy.

### Evaluation of control effectiveness

The differences in the growth trends between predicted and observed cases can be used to benchmark the control of COVID-19 for the prefectures (Additional file 13: Figure S7 and Additional file 14: Figure S8), providing an under-performing or over-performing order. We used the integral of the differences over time to create a total index for control effectiveness. After sorting these indexes, we can identify a list of under-performers and over-performers (Fig. 3c). Indeed, most prefectures in Hubei were prone to be over-performers because of their strong control measures. Prefectures like Zhejiang, Xiaogan and Suizhou were observed as under-performers.

## Discussion

The COVID-19 epidemic broke out in December 2019. China employed almost all the available infectious disease control tools at an unprecedented scale [1] and successfully controlled the epidemic by the end of February 2020. Although importation risks were considered in policy making, the risk-based differentiated need for COVID-19 control in and out of Hubei was inadequately assessed (Fig. 1). The whole of Hubei province was defined as a key area of the epidemic, and a Hubei-specific strategy for COVID-19 control was executed. Other areas followed a non-Hubei control strategy.

The Hubei-specific control strategy consisted of a series of strict stay-at-home measures. We compared non-pharmaceutical interventions between the Hubei and non-Hubei control strategies. The Hubei measures served to enforce adherence to self-quarantine at home directives (Table 1). In contrast, the control strategy executed outside Hubei was typically a series of physical distancing measures (e.g. social distancing) which have been experimentally proven to be effective in delaying and reducing the height of the peak and median epidemic size [7, 19].

Previous studies have documented the strong linear relationship between population movement from Wuhan and the number of infections [20–22]. This study has revealed the effect of applying different control strategies in and out of Hubei. Despite the higher importation risk in Hubei (excluding Wuhan), the epidemic was under control 1 week after COVID-19 containment outside Hubei (Fig. 2a). Both the static and dynamic models showed that the Hubei strategy was a very strong protective factor in contrast with the non-Hubei strategy. This protective effect proved to be time varying. As expected, the higher controlling effect appeared in the earlier dates of the outbreak when the spread was more severe. The protective effect later narrowed gradually and then stabilized (Fig. 3b). By mid-February, the Hubei control strategy obtained 3 times fewer COVID-19 cases compared to the non-Hubei strategy. It’s worth noting that the estimated effects are likely to be conservative because of the introduction of ‘clinically diagnosed cases’ into the analysis, an expanded case definition specifically for Hubei province due to the insufficient testing capacity in the 5th version of the diagnosis and treatment guidelines [23, 24].

The government response is also of interest. Through the statistical modelling, a weak ‘protective’ effect was found for the later implementation of non-Hubei control strategy, and this protective effect disappeared about 2 weeks after implementing the measure (Fig. 3b). This result differs from a previous study [8]. This seemingly unreasonable result has a remarkable interpretation. The Level One response required suspected and confirmed cases to be isolated and reported immediately [6]. During the earliest phase of the epidemic, the virus was diffused, and the response triggered aggressive case and contact identification. This earlier response means that more cases were found earlier and isolated. Therefore, the accelerated response being a ‘risk’ factor is an artifact of improved tracing and disease detection.

Some limitations in our study have been recognized. First, we focused on province-level data outside Hubei unlike the city-level data available for the Hubei area. It is known that the importation risk and daily amount of infections in most cities outside Hubei was incompatible with those in the Hubei area. These differences could confound the effects discovered for the Hubei control strategy. Second, random assignment for sharp discontinuities on cumulative case curves was used to smooth the data. These corrections did not include other considerations of disease progression, such as the incubation period. Early phase fluctuations in case data for the epidemic were at least partly driven by changes in case definitions and adapting health system processes. These issues made it necessary to simplify the assignment process. Third, the cumulative population movement from the epicenter using location-based service data is a simplified estimation of the importation risk at each prefecture. Risk considering case underestimation and reporting delay through probability or mechanistic modelling [25–27] may be another choice. However, extra data collection and complicated calculation may defer the decision making about the epidemic control.

## Conclusions

A risk-based control strategy would improve the effective response for COVID-19 control. Our study shows that the stricter Hubei strategy can achieve better control effectiveness than distancing-focused non-Hubei strategies. These findings highlight the policy impacts and health benefits of precise and differentiated strategies informed by constant monitoring of outbreak risk.

## Supplementary Information

** Additional file 1.** Text. Supplementary methods for data preparation and analysis.

** Additional file 2: Table S1.** Time to execute Level One public health emergency response.

** Additional file 3: Table S2.** Time to execute city shutdown in Hubei.

** Additional file 4: Table S3.** Definitions of suspected and confirmed cases of COVID-19 in six versions implemented before February 29, 2020.

** Additional file 5: Table S4.** Results from the static models.

** Additional file 6: Table S5.** Parameter estimation for the dynamic models.

** Additional file 7: Figure S1.** Comparison of COVID-19 importation risk between Hubei and non-Hubei areas. The risk of COVID-19 importation to each prefecture is defined as their total population inflow from Wuhan until 26 January 2020.

** Additional file 8: Figure S2.** The response timeline of COVID-19 control on human mobility. (a) Provincial population outflow outside Hubei. The flow of each province was represented by the responding BMIs of their capital city. (b) Municipal population outflow in Hubei excluding Wuhan. (c and d) The decline timelines of the outflow BMIs were compared after they were scaled to the values on 22 January. Legends for provincial population flow outside Hubei (a and c) and municipal population flow in Hubei (b and d) are shared, and respectively displayed in (a) and (b). All the colors here followed the response timelines shown in Fig. 1c. BMI: Baidu Mobility Index.

** Additional file 9: Figure S3.** The epidemic curves of COVID-19 in each prefecture by 29 February 2020. Each circular bar on the polar coordinate system represents daily number of reported cases in the prefecture. The grey and red circular lines in bold indicate the dates of Wuhan lockdown and Chinese Lunar New Year. The polar coordinates also show the number of prefectures with COVID-19 case report in parentheses. (a) Provincial epidemic curves outside Hubei. (b) Municipal epidemic curves in Hubei other than Wuhan.

** Additional file 10: Figure S4.** Associations between number of COVID-19 cases, importation risk and control strategy. (a-b) The relationship between the log-transformed importation risk (the total population outflow from Wuhan up to 26 January 2020) and the log-transformed number of confirmed cases by prefectures on 9 February 2020 (a) and 19 February 2020 (b). Circles are prefectures in Hubei; rectangles are prefectures outside Hubei; and the point sizes are proportional to the population density of the prefecture. The linear fitting is done for overall (black), Hubei (red) and non-Hubei (cyan) data. (c-d) The distribution of confirmed cases on 9 February 2020 (c) and 19 February 2020 (d), grouped by governments’ response including response timing and response strategy in a logarithm scale. Samples with insufficient size at the response timing were excluded, such as Qinghai, Tibet and Xiangyang.

** Additional file 11: Figure S5.** Change of the epidemic curves after re-assignment of report date for cases with abnormal fluctuations. They included Shandong and Zhejiang jail cases intensively reported on 20 February 2020 and clinically diagnosed cases in Hubei area due to the amendment of the diagnosis and treatment program of the COVID-19.

** Additional file 12: Figure S6.** Results generated by static models. (a) Change of relative risk over time, generated by the static model II. (b) Change of R^2^ over time, compared between static models.

** Additional file 13: Figure S7.** Predicted versus actual case growth in the prefecture outside Hubei.

** Additional file 14: Figure S8.** Predicted versus actual case growth in the prefecture in Hubei other than Wuhan.

** Additional file 15. **The original case data.

** Additional file 16. **The original population and economic data.

** Additional file 17. **The original travel data.

## Data Availability

All data generated or analyzed during this study are included in this published article and its additional information files. They are all publicly available. Specifically, control measures (Table 1, Additional file 2: Table S1 and Additional file 3: Table S2), case definitions (Additional file 4: Table S3), case data (Additional file 15: Case data), and population and economic data (Additional file 16: Population and economic data) were extracted from local official websites, and travel data (Additional file 17: Travel data) were retrieved through Baidu Qianxi platform (https://qianxi.baidu.com/2020/).

## Abbreviations

COVID-19: Coronavirus disease 2019
dBMI: Daily Baidu Mobility Index
