# Supplementary material for: Evaluation of China’s Hubei control strategy for COVID-19 epidemic: an observational study
Source: BMC Infect Dis. 2021 Aug 16;21:820. doi: 10.1186/s12879-021-06502-z (PMC8366153; doi:10.1186/s12879-021-06502-z)
Supplement: Supplementary file 1 — Additional file 1. Text. Supplementary methods for data preparation and analysis. [file 12879_2021_6502_MOESM1_ESM.docx]

**Supplementary methods for data preparation and analysis**

**S1 Data source**

**S1.1 COVID-19 case data**

Our data of daily infected cases are based on the official data released by the health commission of each province in mainland China. As the first confirmed case outside Wuhan was reported on 19 January, the daily data used in our study are from 19 January to 29 February 2020, including the daily total number of confirmed cases and daily total cumulative number of confirmed cases in each province as well as each city in Hubei province.

**S1.2 Travel data**

Travel data starting 1 January 2020 until 29 February 2020 were collected from Baidu Qianxi platform (<http://qianxi.baidu.com/>). The Baidu Mobility Indexes (BMIs) between cities are provided to represent the mobility volumes and direction, which are divided into the inflow and outflow indexes of individual cities [1-3]. Two pieces of these data were collected. BMIs for population outflow from Wuhan, both in 2020 and 2019 (same lunar date), were first extracted. The outflow BMIs from the capital cities in other provinces of mainland China during the same period in 2020 were also collected. Next, the proportion of human movement from Wuhan in the same period, bound for 31 provinces and 16 non-Wuhan cities in Hubei, were obtained. These proportion data were available for the year 2020.

The daily BMI (dBMI) of population outflow from Wuhan to other provinces and non-Wuhan cities in Hubei was calculated by multiplying the daily outflow index of Wuhan by the corresponding proportion for each area. The cumulative BMI (cBMI) from Wuhan to a target region, a longitudinally accumulated dBMI in a specific time span after COVID-19 outbreak (here, from 1 to 26 January), was generated to measure the importation risk of COVID-19 in the local place.

**S1.3 Control measures**

Because the emergency response is the trigger to activate the systematic measures for the prevention and control of COVID-19 spread, we collected the dates to launch Level One (most urgent) public health emergency response in each province. In Hubei, we used the time to implement the city lockdown to measure the response speed of the local government. The detailed interventions executed in each prefecture were collected from the official websites of local governments and their corresponding health commissions.

**S1.4 Population density and economic data**

Previous study suggested that other factors, such as gross domestic product (GDP) and local population, are correlated with the epidemic [4]. Data on population density and GDP per capita were also collected from 2019 Provincial Statistical Year Books released by the Bureau of Statistics in each prefecture.

**S2 Data analysis**

**S2.1 Re-assignment of abnormal fluctuation of case data**

On 20 February, the new coronavirus was reported to spread in two prisons, one in Shandong and another in Zhejiang. The infectious found in the two jails made up of the 227 newly and intensively reported cases on that day, 200 in Shandong and 27 in Zhejiang. These cases were assumed to be infected several days ago and should have been diagnosed and reported much earlier. To smooth this jumping value on the frequency distribution curve, random technique, which was described below, was used to reassign each with an appropriate report date before 20 February and we called it an ought-to-be report date.

The fifth version of the diagnosis and treatment program of the COVID-19, published by the National Health Commission (NHC) on 5 February [5], introduced a new category named clinically diagnosed cases which referred to as suspected cases with CT scan images showing characteristics of viral pneumonia. This category was only applicable for the hardest-hit Hubei province and led to a sharp uptick in total confirmed cases in the province between 12 and 14 February. And the sixth version amendment canceled this category on 19 February which led to abnormal fluctuation of case report in some prefectures in Hubei. We also assumed the ought-to-be report date of these cases followed the same distribution as those reported earlier before 14 February. And a new report date was reassigned to each using a random rule.

Suppose there was a total of *K* cases having a report delay on day *D*. We assigned each with an ought-to-be report date, one after another. In detail, a case with a report delay on day *D*, denoted by *k* (*k* = 1, 2, 3, …, *K*), was assumed to have an ought-to-be report date $D_{k}^{'}$. We could not know what exactly $D_{k}^{'}$ was but some day before *D*. Because the study time window was in the early phase after COVID-19 outbreak, $D_{k}^{'}$ was simply assumed to have the same distribution with the cases who was reported before *D* and had equal ought-to-be and actual report dates. Let $c_{t}$ be the cumulative number of confirmed cases reported in the prefecture on day *t* (*t* = 1, 2, 3, …, *D*…, 42), where *t* = 1 represented the day on 19 January and *t* = 42 on 29 February. Then $D_{k}^{'}$ followed the cumulative distribution function (CDF)

$$F\left( t \right)=\frac{1}{c_{D}-K}\sum_{T\leq t} {(c}_{t}-K\cdot\mathbf{I}_{\left( T=D \right)})\cdot\mathbf{I}_{\left( T=t \right)}$$

Obviously, it satisfied $F\left( D \right)=1$. In this case, we can draw a random sample from this distribution using the inverse transformation algorithm and generate a presumed ought-to-be report date for the case *S*. That is, sample a random number *u* from a uniform distribution *U*(0, 1), then $D_{k}^{'}=F^{-1}\left( u \right)$. Repeat this process for all the *K* cases. Then a smoothed case distribution was generated as follows:

$$c_{t}^{'}=\left\{ \begin{aligned} &c_{t}+\sum_{k=1}^{K} \mathbf{I}_{\left( D_{k}^{'}\leq t \right)} if t<D \\ &c_{t} if t\geq D \end{aligned} \right.$$

**S2.2 Static model**

The static models were developed to statistically and cross-sectionally investigate the role of interventions during the early phase of the COVID-19 spread across mainland China. These models origin from the gravity model [6], which was inspired by Newton's law of universal gravitation and has been broadly extended and widely applied in the spatial spread of virus and its relationship with mobility and other risk factors [4, 7-11]. When using the gravity model to depict the effect of population outflow from Wuhan on infections in other prefectures $T_{ij}$, the model can be expressed as

$$T_{i1}=Km_{i}m_{1}f\left( d_{i1} \right)$$

where $K$ is a constant, $m_{i}$ and $m_{1}$ are the masses related to the number of trips attracted by prefecture $i$ or leaving Wuhan ($j=1$), and $f\left( d_{i1} \right)$ is called a friction factor or deterrence function [12]. To control other possible confounding factors like per capita GDP and population density, we also introduced them into the mass variables. Because we fix $j=1$ for Wuhan, the mass variables about Wuhan can be dropped. The containment strategies (Hubei or non-Hubei), action taken date (23, 24, or 25 January) and their interaction are modelled as friction factors. All independent variables here are given in an exponent form. Then the model can be parameterized as

| $y_{i}=c{\cdot e}^{\sum_{k}^{m} \beta_{k}x_{ki}}e^{\lambda_{1}\cdot I_{Hubei}+\lambda_{2}\cdot D_{response}+I_{Hubei}*D_{response}}$ | (1) |
| --- | --- |

where $y_{i}$ is the number of cumulative confirmed cases in prefecture *i*, $x_{1i}$ is the cBMI outflow from Wuhan between 1 to 26 January to prefecture *i*, $x_{2i}$ is per capita GDP of prefecture *i*, $x_{3i}$ is the population density of prefecture *i*, *m* is the number of explained variables involved, *c* and $\beta_{j}$ are parameter estimates. If taking the logarithm on both sides, model (1) has the form of a generalized model:

$\log\left( y_{i} \right)=\log\left( c \right)+\sum_{j}^{m} \beta_{j}x_{ji}+\lambda_{1}\cdot I_{Hubei}+\lambda_{2}\cdot D_{response}+I_{Hubei}*D_{response}$ (2)

And model (2) becomes a Negative Binomial regression model under the assumption that $y_{i}$ follows a Negative Binomial distribution. When the only one independent variable of cumulative population outflow from Wuhan is involved, the statistical model equals to the linear correlation analysis between total confirmed cases and the importation risk from Wuhan, both in a logarithm scale.

To eliminate differences in units in modelling, the data were first pre-processed and normalized. For example, we took the logarithm for $x_{1i}$ (that is, the cBMI) based on the detective association in exploratory analysis, $x_{i1}^{'}=\log\left( x_{1i} \right)$, then standardized it as $x_{i1}^{''}=\frac{x_{i1}^{'}-Mean}{Std}$. To get a consistent estimation throughout the context, the Levenberg-Marquardt (LM) algorithm, combining the advantages of the steepest gradient algorithm and the Newton algorithm [12, 13], was used to obtain the nonlinear least square estimation for the parameters in model (1). This algorithm has been integrated in minpack.lm package in R software [14] and models were fitted using the nlsLM function in the package.

To observe the effect generated by COVID-19 control, we first fit the model (1) with three risk factors including cBMI outflow from Wuhan (from 1 to 26 January) to our study prefectures, per capita GDP and population density in each destination. The variables which provide no evidence about the predictive ability with *P* > 0.05 was excluded before next-step analysis. Then the containment strategy was included into models and changes of Bayesian Information Criteria (BIC) and *R*^2^ were evaluated.

**S2.3 Dynamic model**

The Cox proportional hazards model to integrate the independent variables with a growth function:

$h\left( t | x_{i} \right)=h_{0}\left( t \right)e^{\sum_{j}^{m} \beta_{j}x_{ji}} e^{\lambda_{1}\cdot I_{Hubei}+\lambda_{2}\cdot D_{measure}+{\lambda_{2}\cdot I}_{Hubei}*D_{measure}}$

where $h\left( t | x_{i} \right)$ is the hazard function describing the number of cumulative confirmed cases at time *t* given an population outflow from Wuhan to prefecture *i* and other variables; $h_{0}\left( t \right)$ is the underlying baseline hazard function with *t* =1 starting from 26 January; $x_{i}=\left\{ x_{1i}, x_{2i}, \cdots, x_{mi} \right\}$are the realized values of the covariates for prefecture *i*; and the other notation is the same as for the static model. The logistic function is used as the sigmoidal function, with its functional curve growing exponentially initially and saturating at the later stage. Then, the dynamic model becomes

$$h\left( t | x_{i} \right)=\frac{\alpha}{1+e^{-\gamma t+\omega}}e^{\sum_{j}^{m} \beta_{j}x_{ji}} e^{\lambda_{1}\cdot I_{Hubei}+\lambda_{2}\cdot D_{response}+\lambda_{3}\cdot I_{Hubei}*D_{response}}$$

We explored and improved the dynamic step by step and following models were investigated:

Dynamic model I:

$$h\left( t | x_{i} \right)=\frac{\alpha}{1+e^{-\gamma t+\omega}}e^{\sum_{j}^{m} \beta_{j}x_{ji}} e^{\lambda_{1}\cdot I_{Hubei}}$$

Dynamic model II:

$$h\left( t | x_{i} \right)=\frac{\alpha}{1+e^{-\gamma t+\omega}}e^{\sum_{j}^{m} \beta_{j}x_{ji}} e^{\lambda_{1}\cdot I_{Hubei}+\lambda_{2}\cdot D_{response}+\lambda_{3}\cdot I_{Hubei}*D_{response}}$$

Dynamic model III:

$$h\left( t | x_{i} \right)=\frac{\alpha}{1+e^{-\gamma t+\omega}}e^{\sum_{j}^{m} \beta_{j}x_{ji}} e^{{(\lambda}_{1a}+\lambda_{1b}\cdot t+\lambda_{1c}\cdot t^{2})\cdot I_{Hubei}+\lambda_{2}\cdot D_{response}+\lambda_{3}\cdot I_{Hubei}*D_{response}}$$

Dynamic model IV:

$$h\left( t | x_{i},T \right)=\frac{\alpha}{1+e^{-\gamma t+\omega}}e^{\sum_{j}^{m} \beta_{j}x_{ji}} e^{{(\lambda}_{1a}+\lambda_{1b}\cdot t+\lambda_{1c}\cdot t^{2})\cdot I_{Hubei}+\lambda_{2}\cdot I_{t\leq T}\cdot D_{response}+\lambda_{3}\cdot I_{Hubei}*D_{response}}$$

**References**

1. Liu K, Ai S, Song S, Zhu G, Tian F, Li H, et al. Population movement, city closure in Wuhan and geographical expansion of the 2019-nCoV pneumonia infection in China in January 2020. Clin Infect Dis. 2020:ciaa422.

2. Chinazzi M, Davis JT, Ajelli M, Gioannini C, Litvinova M, Merler S, et al. The effect of travel restrictions on the spread of the 2019 novel coronavirus (COVID-19) outbreak. Science. 2020; 368:395-400.

3. Chan JF, Yuan S, Kok KH, To KK, Chu H, Yang J, et al. A familial cluster of pneumonia associated with the 2019 novel coronavirus indicating person-to-person transmission: a study of a family cluster. Lancet. 2020; 395:514-23.

4. Jia JS, Lu X, Yuan Y, Xu G, Jia J, Christakis NA. Population flow drives spatio-temporal distribution of COVID-19 in China. Nature. 2020; 582:389–94.

5. China's State Council Information Office. Fighting COVID-19: China in Action. The State Council Information Office of the People's Republic of China. 2020. Available:http://english.www.gov.cn/news/topnews/202006/07/content_WS5edc559ac6d066592a449030.html. Accessed 15 Sep 2000.

6. Zipf GK. The P1 P2/D Hypothesis: On the Intercity Movement of Persons. American Sociological Review. 1946; 11:677–86.

7. Wesolowski A, Qureshi T, Boni MF, Sundsoy PR, Johansson MA, Rasheed SB, et al. Impact of human mobility on the emergence of dengue epidemics in Pakistan. Proc Natl Acad Sci U S A. 2015; 112:11887-92.

8. Bengtsson L, Lu X, Thorson A, Garfield R, von Schreeb J. Improved response to disasters and outbreaks by tracking population movements with mobile phone network data: a post-earthquake geospatial study in Haiti. PLoS Med. 2011; 8:e1001083.

9. Viboud C, Bjornstad ON, Smith DL, Simonsen L, Miller MA, Grenfell BT. Synchrony, waves, and spatial hierarchies in the spread of influenza. Science. 2006; 312:447-51.

10. Balcan D, Colizza V, Goncalves B, Hu H, Ramasco JJ, Vespignani A. Multiscale mobility networks and the spatial spreading of infectious diseases. Proc Natl Acad Sci U S A. 2009; 106:21484-9.

11. Barrios JM, Verstraeten WW, Maes P, Aerts JM, Farifteh J, Coppin P. Using the gravity model to estimate the spatial spread of vector-borne diseases. Int J Environ Res Public Health. 2012; 9:4346-64.

12. Barbosa H, Barthelemy M, Ghoshal G, James CR, Lenormand M, Louail T, et al. Human mobility: Models and applications. Phys Rep. 2018; 734:1-74.

13. Song CM, Koren T, Wang P, Barabasi AL. Modelling the scaling properties of human mobility. Nat Phys. 2010; 6:818-23.

14. R Core Team. R: A language and environment for statistical computing. R Foundation for Statistical Computing, Vienna, Austria. R Core Team. 2020. Available: <https://www.R-project.org/>. Accessed 15 Feb 2020.
