# Supplementary figures and images for: Evaluation of China’s Hubei control strategy for COVID-19 epidemic: an observational study
Source: BMC Infect Dis. 2021 Aug 16;21:820. doi: 10.1186/s12879-021-06502-z (PMC8366153; doi:10.1186/s12879-021-06502-z)

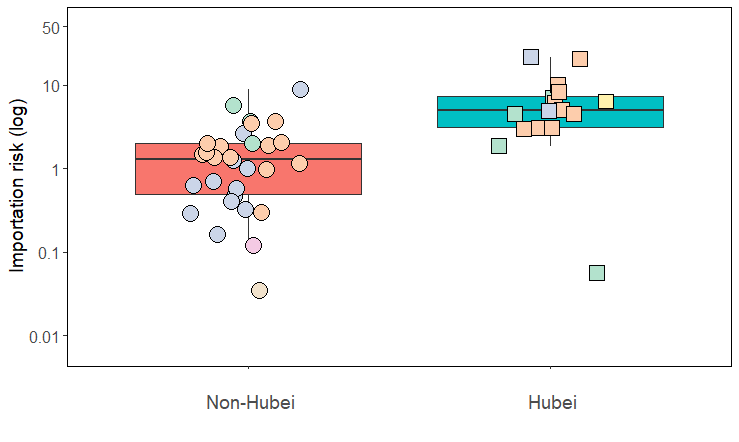

Supplement: Supplementary file 7 — Additional file 7: Figure S1. Comparison of COVID-19 importation risk between Hubei and non-Hubei areas. The risk of COVID-19 importation to each prefecture is defined as their total population inflow from Wuhan until 26 January 2020. [file 12879_2021_6502_MOESM7_ESM.tiff]

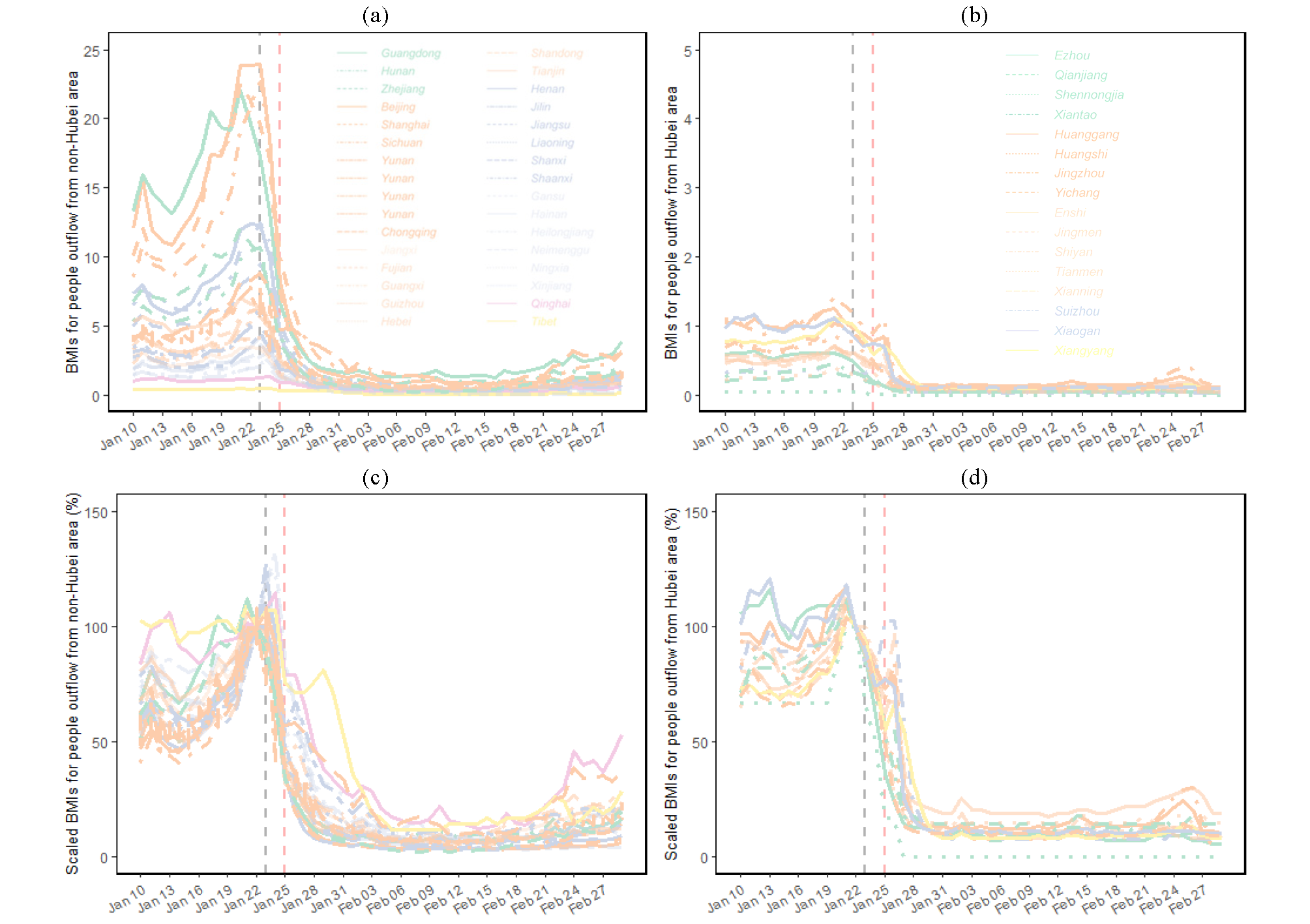

Supplement: Supplementary file 8 — Additional file 8: Figure S2. The response timeline of COVID-19 control on human mobility. (a) Provincial population outflow outside Hubei. The flow of each province was represented by the responding BMIs of their capital city. (b) Municipal population outflow in Hubei excluding Wuhan. (c and d) The decline timelines of the outflow BMIs were compared after they were scaled to the values on 22 January. Legends for provincial population flow outside Hubei (a and c) and municipal population flow in Hubei (b and d) are shared, and respectively displayed in (a) and (b). All the colors here followed the response timelines shown in Fig. 1c. BMI: Baidu Mobility Index. [file 12879_2021_6502_MOESM8_ESM.tiff]

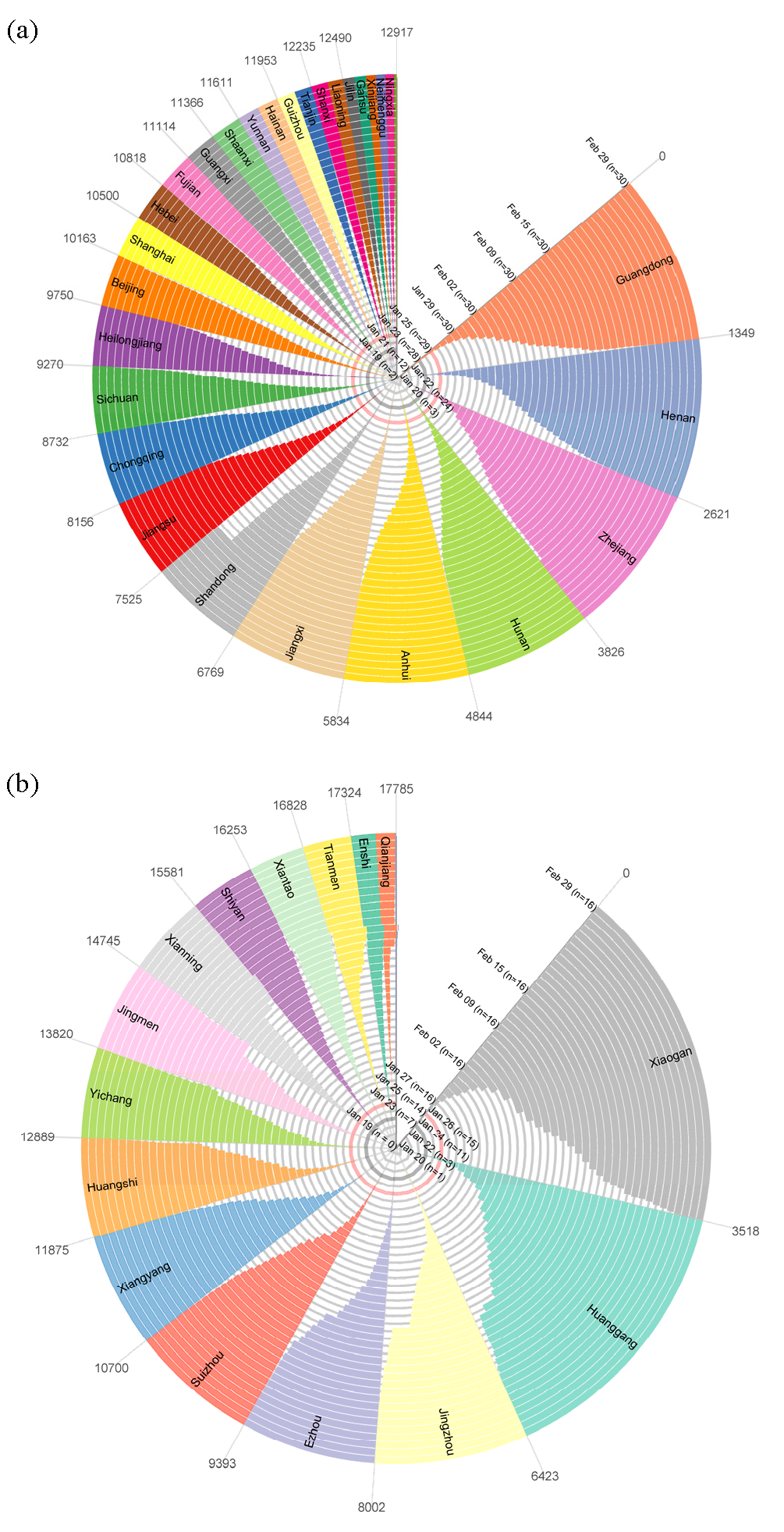

Supplement: Supplementary file 9 — Additional file 9: Figure S3. The epidemic curves of COVID-19 in each prefecture by 29 February 2020. Each circular bar on the polar coordinate system represents daily number of reported cases in the prefecture. The grey and red circular lines in bold indicate the dates of Wuhan lockdown and Chinese Lunar New Year. The polar coordinates also show the number of prefectures with COVID-19 case report in parentheses. (a) Provincial epidemic curves outside Hubei. (b) Municipal epidemic curves in Hubei other than Wuhan. [file 12879_2021_6502_MOESM9_ESM.tiff]

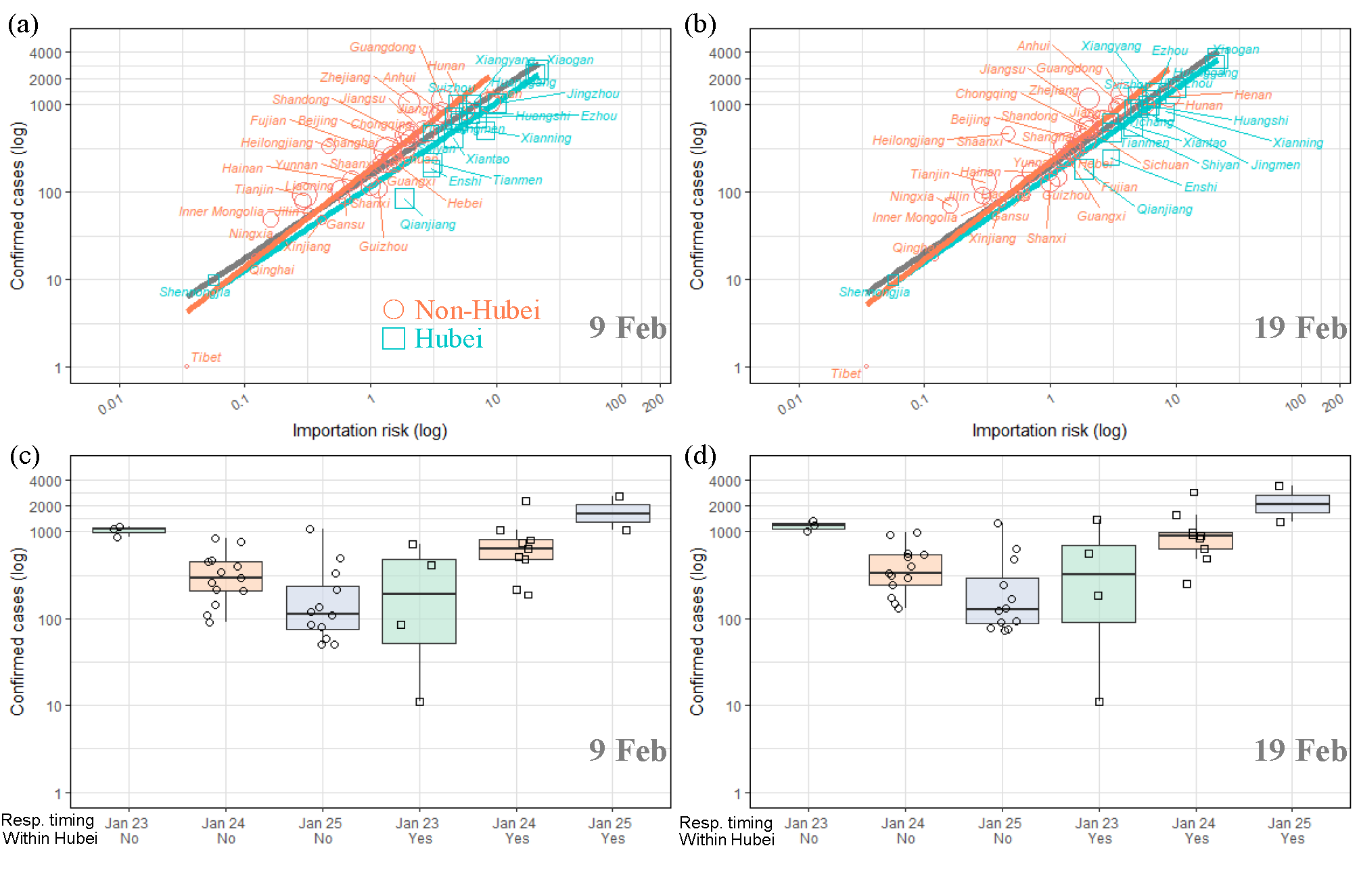

Supplement: Supplementary file 10 — Additional file 10: Figure S4. Associations between number of COVID-19 cases, importation risk and control strategy. (a-b) The relationship between the log-transformed importation risk (the total population outflow from Wuhan up to 26 January 2020) and the log-transformed number of confirmed cases by prefectures on 9 February 2020 (a) and 19 February 2020 (b). Circles are prefectures in Hubei; rectangles are prefectures outside Hubei; and the point sizes are proportional to the population density of the prefecture. The linear fitting is done for overall (black), Hubei (red) and non-Hubei (cyan) data. (c-d) The distribution of confirmed cases on 9 February 2020 (c) and 19 February 2020 (d), grouped by governments’ response including response timing and response strategy in a logarithm scale. Samples with insufficient size at the response timing were excluded, such as Qinghai, Tibet and Xiangyang. [file 12879_2021_6502_MOESM10_ESM.tiff]

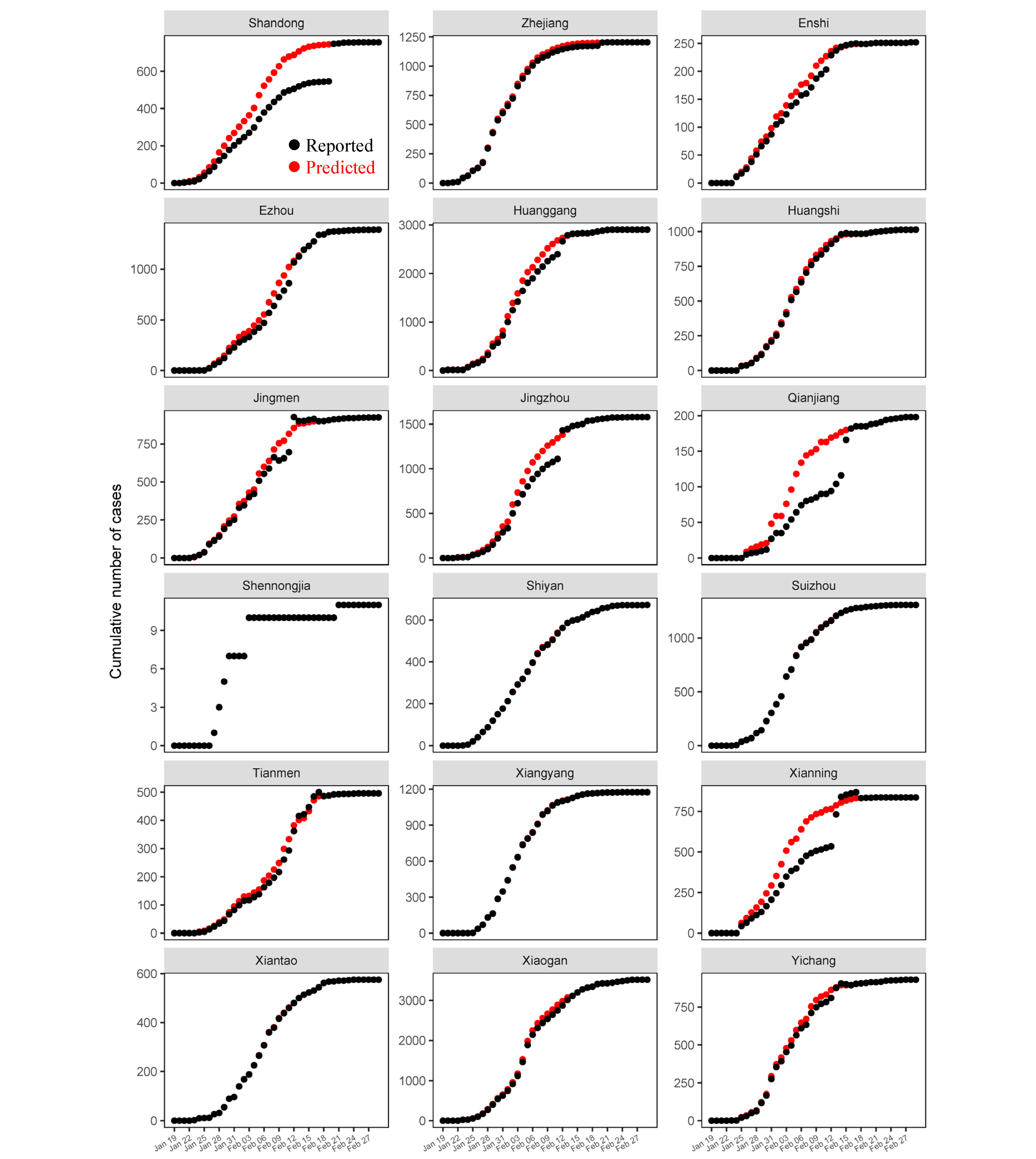

Supplement: Supplementary file 11 — Additional file 11: Figure S5. Change of the epidemic curves after re-assignment of report date for cases with abnormal fluctuations. They included Shandong and Zhejiang jail cases intensively reported on 20 February 2020 and clinically diagnosed cases in Hubei area due to the amendment of the diagnosis and treatment program of the COVID-19. [file 12879_2021_6502_MOESM11_ESM.tiff]

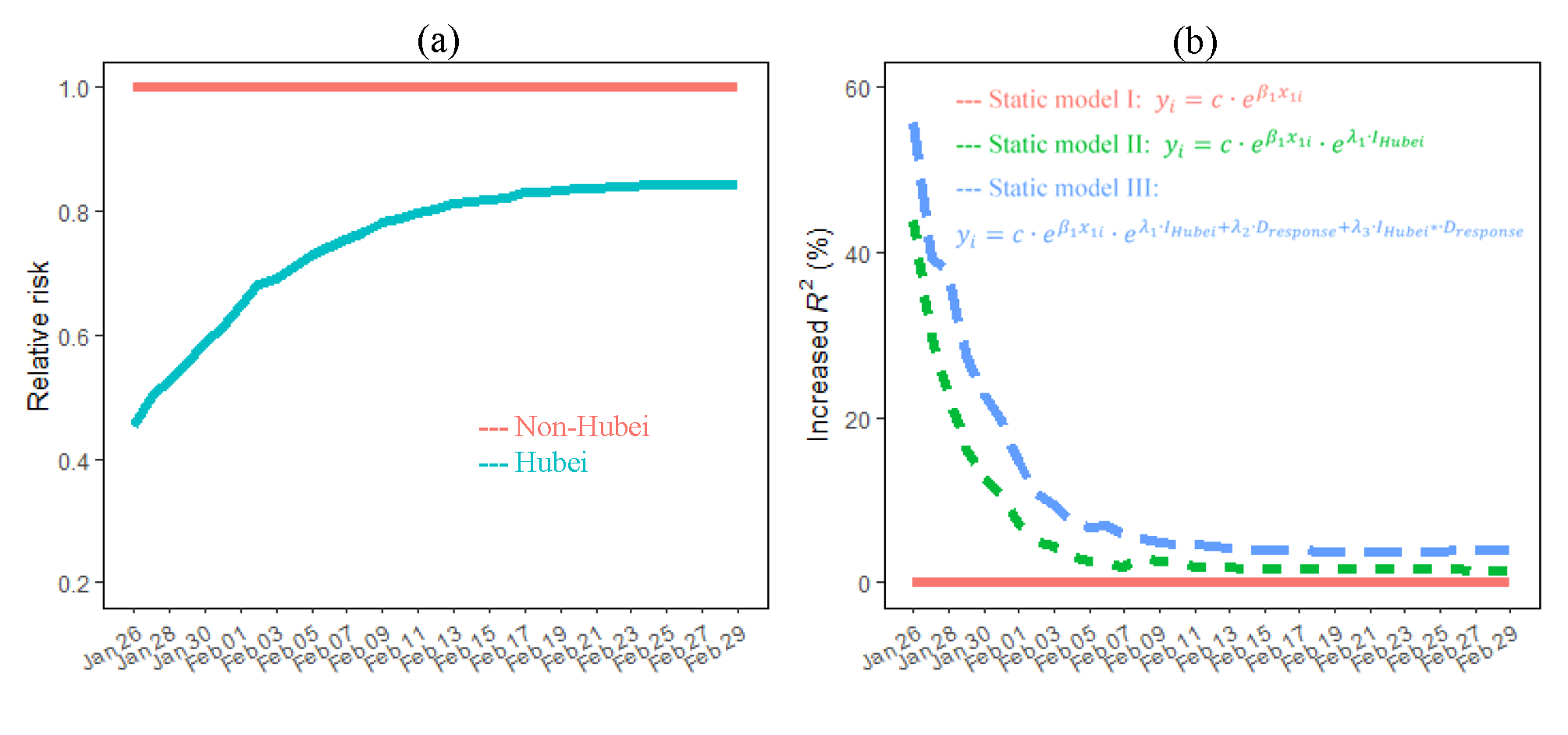

Supplement: Supplementary file 12 — Additional file 12: Figure S6. Results generated by static models. (a) Change of relative risk over time, generated by the static model II. (b) Change of R2 over time, compared between static models. [file 12879_2021_6502_MOESM12_ESM.tiff]

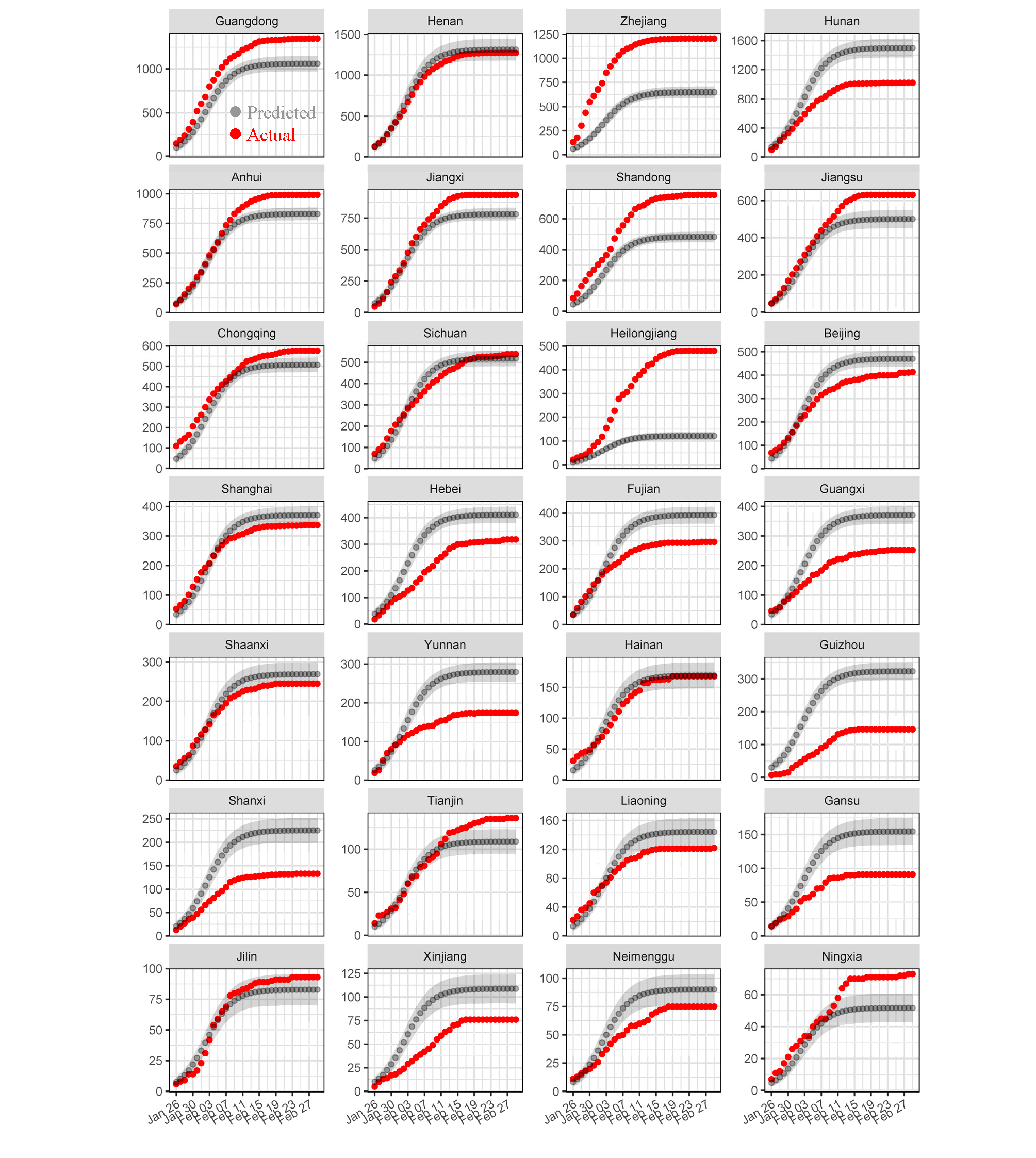

Supplement: Supplementary file 13 — Additional file 13: Figure S7. Predicted versus actual case growth in the prefecture outside Hubei. [file 12879_2021_6502_MOESM13_ESM.tif]

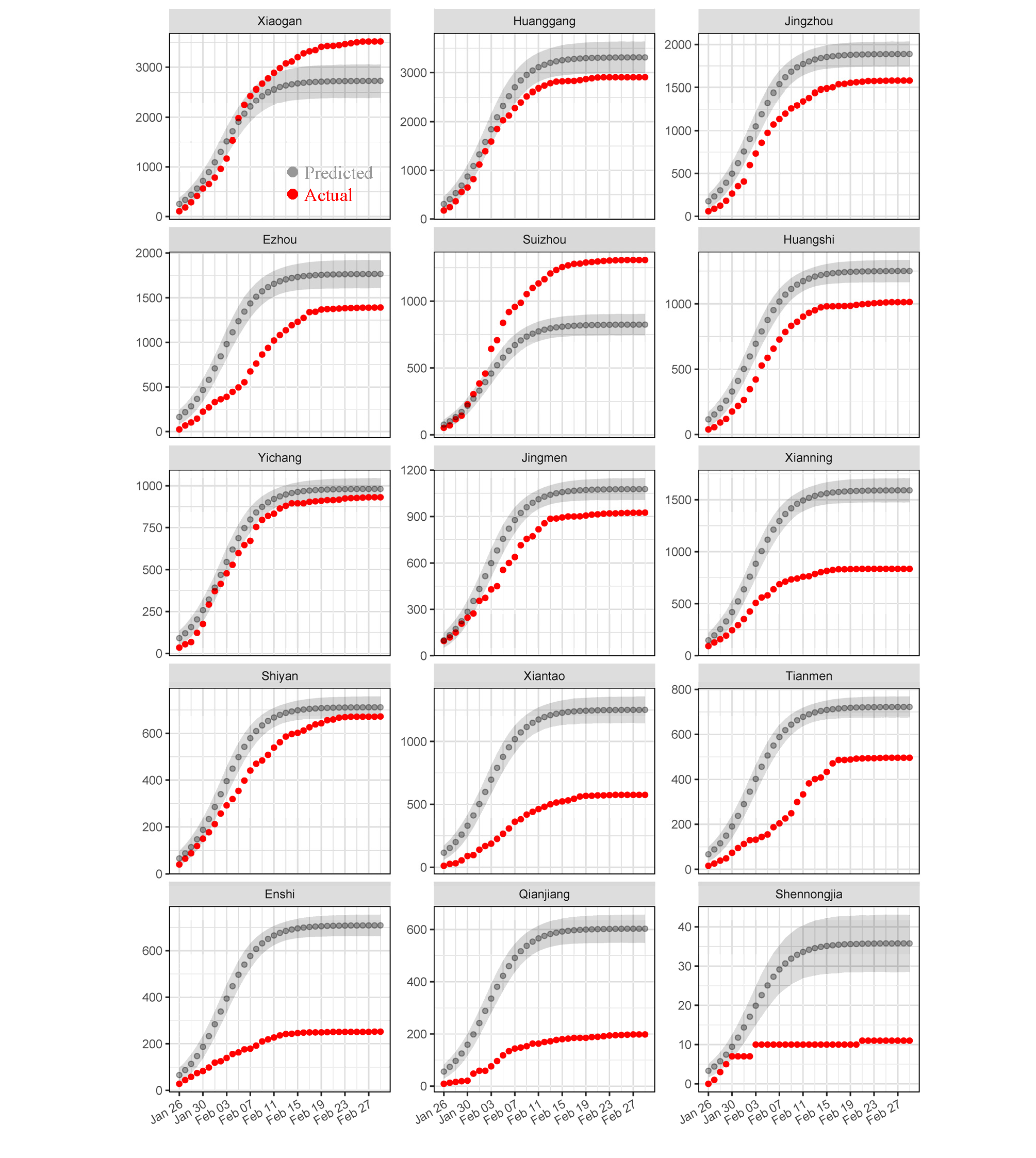

Supplement: Supplementary file 14 — Additional file 14: Figure S8. Predicted versus actual case growth in the prefecture in Hubei other than Wuhan. [file 12879_2021_6502_MOESM14_ESM.tif]
